# *Venatorbacter cucullus* gen. nov sp. nov a novel bacterial predator

**DOI:** 10.1038/s41598-021-00865-8

**Published:** 2021-11-01

**Authors:** Ahmed Saeedi, Nicola J. Cummings, Denise McLean, Ian F. Connerton, Phillippa L. Connerton

**Affiliations:** 1 Division of Microbiology, Brewing and Biotechnology, School of Biosciences, University of Nottingham, Sutton Bonington Campus, Loughborough, LE12 5RD UK; 2 Nanoscale and Microscale Research Centre, University of Nottingham, Nottingham, NG7 2RD UK

**Keywords:** Ecology, Evolution, Microbiology

## Abstract

A novel Gram-stain negative, aerobic, halotolerant, motile, rod-shaped, predatory bacterium ASxL5^T^, was isolated from a bovine slurry tank in Nottinghamshire, UK using *Campylobacter hyointestinalis* as prey. Other *Campylobacter* species and members of the *Enterobacteriaceae* were subsequently found to serve as prey. Weak axenic growth on Brain Heart Infusion agar was achieved upon subculture without host cells. The optimal growth conditions were 37 °C, at pH 7. Transmission electron microscopy revealed some highly unusual morphological characteristics related to prey availability. Phylogenetic analyses using 16S rRNA gene sequences showed that the isolate was related to members of the *Oceanospirillaceae* family but could not be classified clearly as a member of any known genus. Whole genome sequencing of ASxL5^T^ confirmed the relationship to members the *Oceanospirillaceae*. Database searches revealed that several ASxL5^T^ share 16S rRNA gene sequences with several uncultured bacteria from marine, and terrestrial surface and subsurface water. We propose that strain ASxL5^T^ represents a novel species in a new genus. We propose the name *Venatorbacter cucullus* gen. nov., sp. nov. with ASxL5^T^ as the type strain.

## Introduction

A predatory bacterium is one that demonstrates the ability to pursue and kill other living bacteria to obtain biosynthetic materials and energy^1^. This is distinct from the universal recycling of the nutrients from dead microorganisms and from parasitic interactions where bacteria form close associations with their hosts without killing them. Predatory bacteria have evolved diverse life cycles to exploit abundant food sources in the niches where they are found, for example in marine habitats^2^. They are a taxonomically diverse group connected only by their unique bactericidal life cycle^1^. Examples of predatory bacteria are found in several different phyla including: Proteobacteria, Bacteroidetes and Chloroflexi^3^. However, the most well-studied predatory bacteria are, *Bdellovibrio* and *Bdellovibrio*-and-like organisms (BALOs^4^). Predatory bacteria are promising sources of new bioactive compounds and antimicrobials^5^.

Predatory bacteria are suggested to enhance microbial diversity, and have positive effects on ecosystem health, productivity, and stability^6^. Despite these positive attributes, there are few studies of new predatory bacteria because of difficulties in culturing the bacteria, and the need for careful observation of cellular interactions in order to understand their complex lifecycles. This information is not readily available from in silico analysis.

In an era of increased antimicrobial resistance novel strategies such as the use of bacteriophage and predatory bacteria, that target bacterial pathogens, are being investigated^7,8^. The ASxL5^T^ bacterium was isolated from cattle slurry collected from the University of Nottingham Dairy Centre, Nottinghamshire, in 2019 using techniques for phage isolation^9^. The aim of the investigation was to isolate organisms that had potential as biocontrol agents. *Campylobacter hyointestinalis*, a zoonotic pathogen that is increasingly associated with enteric disease in humans^10^, was prevalent in the slurry and was used as a target host.

## Results

### ASxL5^T^ is a predatory bacterium with unusual cell morphology

**Figure 1:**
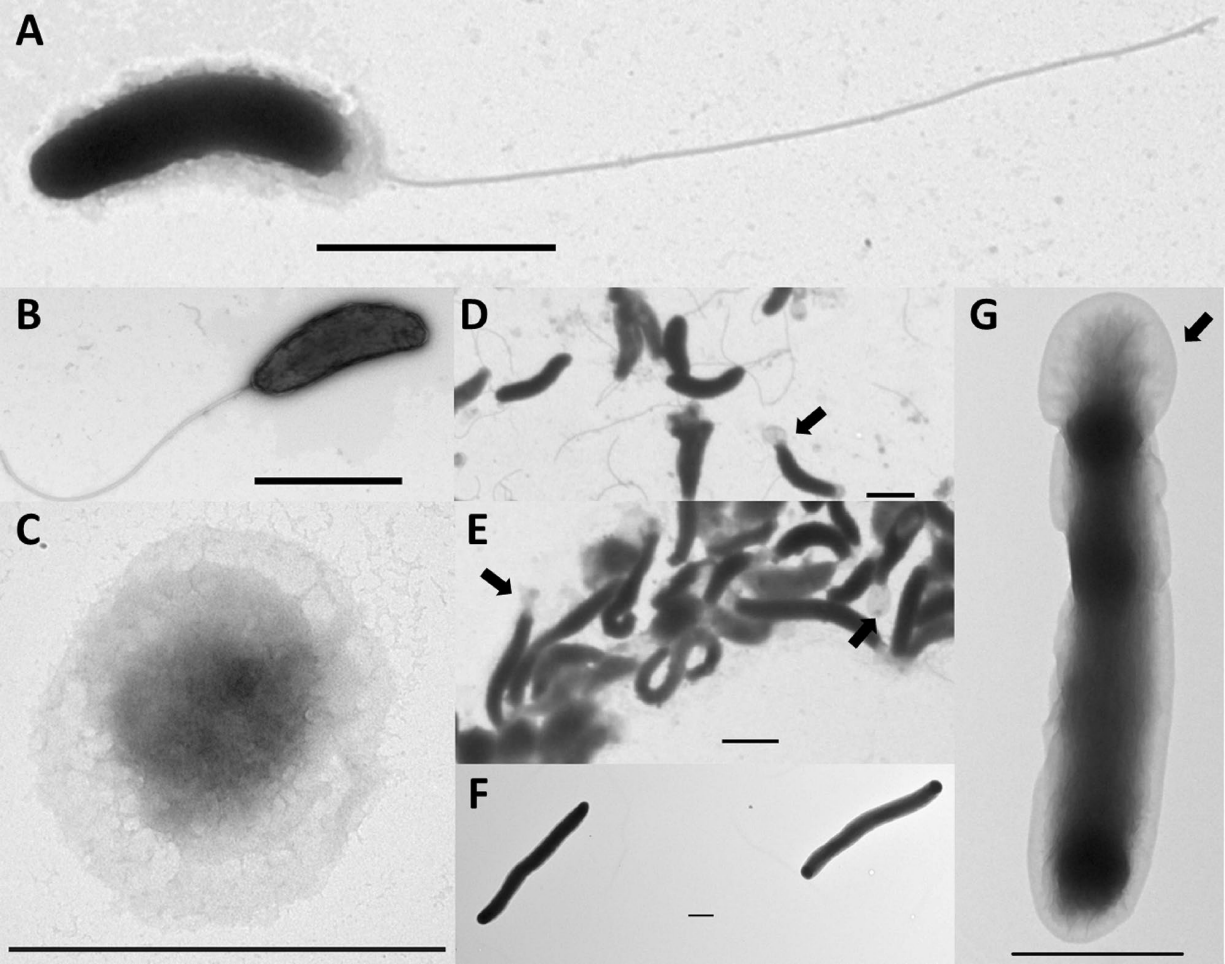
TEM of ASx5L^T^ showing: (**A**) ASx5L^T^ showing long polar flagellum; (**B**) typical ASx5L^T^ cell; (**C**) coccal ASx5L^T^ cell following prolonged incubation without nutrients; (**D**) group of ASx5L^T^ cells showing unusual apical structure indicated with arrow; (**E**) group of ASx5L^T^ cells incubated with *Campylobacter* prey showing increased cell length compared with those grown without prey (**D**) also showing apical structures; (**F**) large filamentous aflagellate, ASx5L^T^ cells, following incubation with *E. coli* prey; (**G**) single ASx5L^T^ cell following incubation with *E. coli* showing unusual apical structure. Bar represents 1 μm.

The ASxL5^T^ bacterium was isolated from bovine slurry due to the observation that it formed plaques on *C. hyointestinalis* lawns similar to those produced by bacteriophage. It was an unexpected finding because part of the phage isolation procedure involved filtration through a 0.2 µm filter designed to remove bacterial cells. Microscopic examination of the material extracted from the plaques revealed small Gram-stain negative curved rod-shaped bacteria that did not accumulate polyhydroxybutyrate (PHB). Axenic culture was achieved independent of prey cells on rich solid media such as Brain Heart Infusion agar (BHI) and Blood agar (BA), with weak growth that improved on subculture using heavy inocula. Growth occurred equally well under microaerobic (7% v/v oxygen) and atmospheric oxygen conditions but not in an anaerobic atmosphere. Colonies were small reaching 2 mm in diameter after 72 h and were beige, translucent, circular, convex and shiny. Standard biochemical tests were hampered as ASxL5^T^ could not be reliably cultured in liquid medium suggesting a complex life cycle with possible dependence on biofilm formation. However, plate suspensions demonstrated ASxL5^T^ was aerobic, oxidase and catalase positive and able to tolerate 5% NaCl. ASxL5^T^ was resistant to streptomycin 10 µg, but sensitive to all other antibiotics tested. The ASxL5^T^ bacterial cells were examined by TEM (Fig. 1). When grown without prey cells on BA, the ASxL5^T^ cells were small, curved bacteria with an average length of 1.63 μm (± 0.4) and width of 0.37 μm (± 0.08), with a single long (up to 5 μm) polar flagellum. Approximately 1.6% of cells appeared to have a width of less than 0.2 μm, which would allow passage through a filtration device. An unusual structural extension resembling a cowl (latin *cucullus*), was observed at the apex of some cells (see arrows in Fig. 1D,E,G). This appeared to be composed of excess outer membrane, possibly due to a rapid reduction in size of the periplasmic envelope, with the outer membrane remaining intact, giving a “baggy” appearance. Prolonged incubation of ASxL5^T^ without nutrients (in PBS), at 4 °C, resulted in most, but not all, of the cells exhibiting coccal morphology (Fig. 1C). When ASxL5^T^ was grown for 48 h with *C. jejuni* as prey, the mean cell sizes were significantly longer and narrower, than cells grown without host (Table 1 and Fig. 1E). In contrast when ASxL5^T^ was grown for 48 h with *E. coli* as prey, the mean cell sizes were longer and wider than when grown without prey (Table 1), and the cell length was variable, often showing filamentation (Fig. 1F). ASxL5^T^ cells showed a complete absence of flagella when incubated for 48 h with either *C. jejuni* or *E. coli* as prey. Observations of the variation in cell size according to presence, absence, and type of prey of ASxL5^T^ are summarised in Table 1.

**Table 1:** Variation in size of ASx5L^T^ with presence, absence, and prey type determined from TEM images.

Growth condition (48 h at 37 °C)	Mean cell dimensions	Difference from BA (ANOVA p-value)
**Grown on BA**		
Length	1.63 µm (± 0.42)	
Width	0.37 µm (± 0.08)	
**Incubated with *C. jejuni***		
Length	2.09 µm (± 0.69)	0.0003
Width	0.30 µm (± 0.06)	4 × 10^–15^
**Incubated with *E. coli***		
Length	4.99 µm (± 2.45)	0.00016
Width	0.63 µm (± 0.11)	2 × 10^–15,^

### The genome sequence of ASxL5^T^ reveals a relationship with marine bacteria

**Figure 2:**
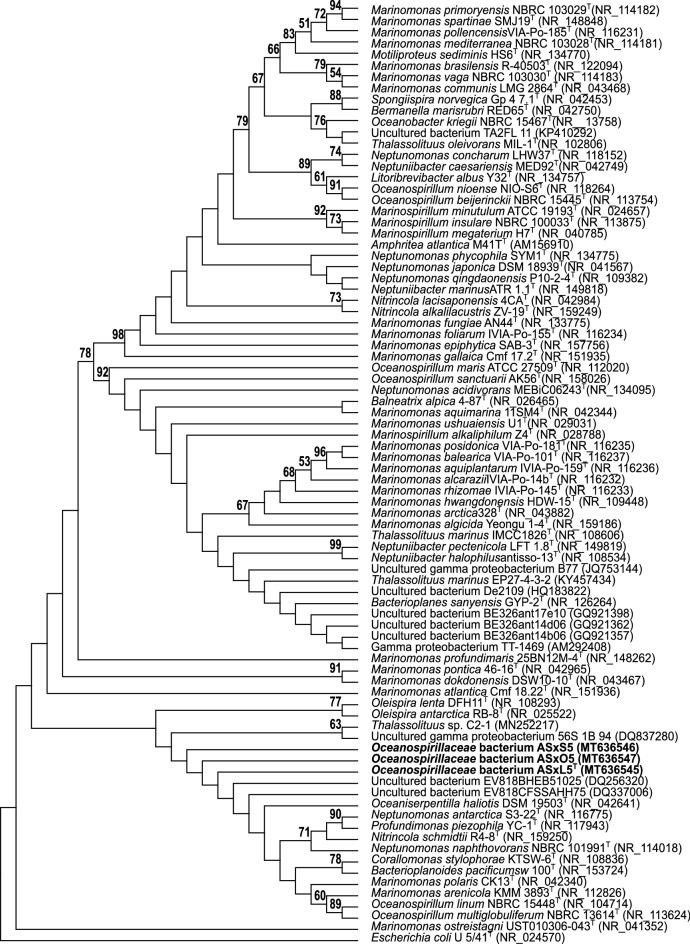
Phylogenetic tree using 16S rRNA gene sequences highlighting the position of ASxL5^T^, ASxO5 and ASxS5strains (emboldened) relative to uncultured and strains of marine bacteria genera within the family *Oceanospirillaceae*. Genbank accession numbers follow the strain name in parenthesis. Sequences were aligned using ClustalW and the phylogenic relationship inferred using the Maximum Likelihood method with the Tamura-Nei model, with 1000 bootstrap replicates, within the MEGA X program. Numbers on branches indicate bootstrap replicate values greater than 50%. *Escherichia coli* U/541^T^ was used as an outgroup.

**Figure 3:**
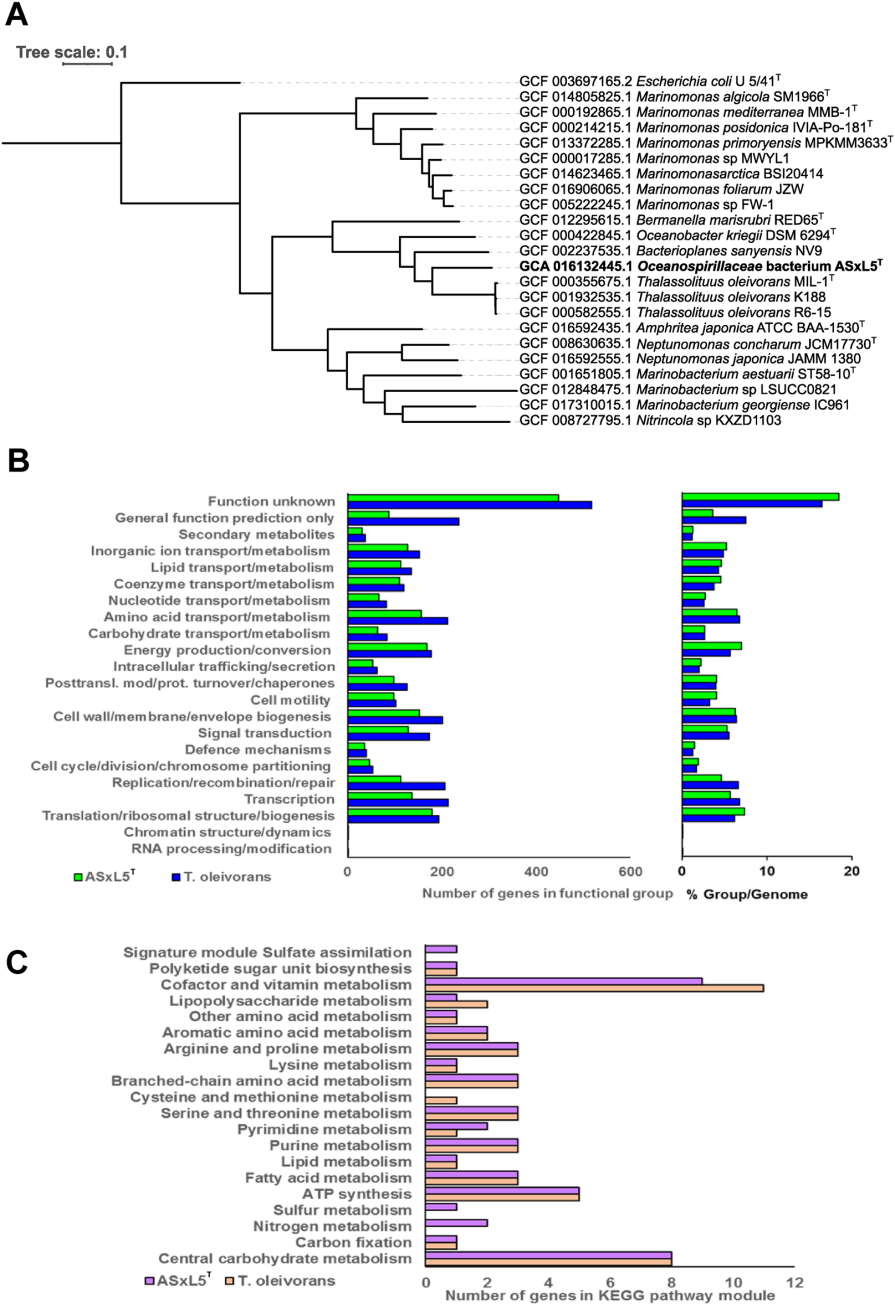
(**A**) Genome-based phylogenetic tree showing the relationship of *Oceanospirillaceae* bacterium ASxL5^T^ to closest relatives in the *Oceanospirillaceae* family with *E. coli* U 5/41^T^ as an outgroup. (**B**) Functional class distribution of predicted genes according to the clusters of orthologous groups (COG) of proteins of ASx5L^T^ compared to *T. oleivorans* MIL-1^T^. Left panel shows the number of genes per functional COG category for each genome. The right panel shows the percentage of the genome that each functional COG group comprises. (**C**) Analysis of complete KEGG (Kyoto encyclopedia of genes and genomes) module pathways for ASxL5^T^ compared to *T. oleiverans* MIL-1^T^.

Determination of the 16S rRNA gene sequence (accession number MT636545.1) enabled database searches to establish the sequence resembled those in the class *Gammaproteobacteria* and were most closely aligned with marine bacteria in the family *Oceanospirillaceae* (Fig. 2) with members of the genera *Thalassolituus* and *Oceanobacter* the nearest relatives. The 16S rRNA gene sequences were notably diverged from predatory bacteria belonging to the family *Bdellovibrionaceae* (*Deltaproteobacteria*). The pairwise alignments for *B. bacteriovorus* HD100^T^ (type strain, DSM 50701) and *B. bacteriovorus* DM11A were 48.4% and 47.7% identity, and for *B. exovorus* JSS 46.7% identity. The ASxL5^T^ bacterium had 3 copies of the 16S rRNA genes with two being identical to each other and the third differing by 3 bases. Two further predatory bacterial isolates from the same location with similar morphology and phenotypic characteristics (ASx5S and ASx5O; 16S rRNA gene accession numbers MT636546.1 and MT636547.1 respectively) were not identical, but clustered with ASxL5^T^ and uncultured bacterial database sequences, separate from other genera in the *Oceanospirillaceae* (Fig. 2). The whole genome sequence of ASxL5^T^ was determined and deposited in the NCBI database under the accession number CP046056. The genome of ASxL5^T^ consisted of a single circular chromosome of 2,831,152 bp with a G + C ratio of 56.1%. The genome sequence contained 2653 CDSs (total), of which 2567 were predicted to encode proteins, and of these 1596 could be assigned a putative function (60.2%). The genome contained 67 RNA encoding genes comprising of 9 rRNAs (3 each 5S, 16S and 23S) together with 57 tRNAs. The genomic characteristics of ASxL5^T^ were compared to the available genomes of the type strains of the closest relatives identified from the 16S rRNA gene sequences (Table 2). All available *Thalassolituus* genomes were compared with ASxL5^T^ using amino acid identity (AAI). The closest available genome sequence (incomplete) determined by AAI was that of *Thalassolituus* sp. C2-1 (accession NZ_VNIL01000001). This strain was isolated from a deep-sea sediment of the Mariana Trench, but no phenotypic information regarding this strain is available for comparison at present. This organism has a much larger genome at 4.36 Mb compared to 2.82 Mb for ASxL5^T^. The average genome size for a member of the order *Oceanospirillales* is approximately 4.16 Mb (± 1.1; n = 92 complete reference genomes surveyed from https://www.ncbi.nlm.nih.gov/assembly), so the genome of ASxL5^T^ is quite small compared to other members of the order. A genome-based estimated maximum-likelihood phylogenetic tree (Fig. 3A) was generated with GToTree 1.5.54 utilizing aligned and concatenated amino acid sequences of 172 single-copy genes specific to *Gammaproteobacteria* ^11–18^. This analysis demonstrated a close relationship to *Thalassolituus, Bacterioplanes,* and *Oceanobacter* genera. However, these data indicate that ASxL5^T^ is distinct from its relatives in the *Oceanospirillaceae* for which genomic sequence data are available.

**Table 2:** Genomic comparison of ASx5L^T^ with type strains of related genera.

Genome	Accession no.	Genome size (Mb)	G + C ratio	16S RNA identity	AF	ANI	AAI
ASxL5^T^	CP046056	2.82	56.1	100	100	100	100
*Thalassolituus oleivorans* MIL-1^T^	HF680312	3.9	46.6	95.03	0.421	71.86	67.61
*Bacterioplanes sanyensis* KCTC 32220^T^	BMYY01000001	3.83	53.4	94.64	0.394	72.87	66.78
*Oceanobacter kriegii* DSM 6294^T^	NZ_AUGV00000000	4.5	55.3	94.14	0.327	74.10	53.78
*Marinomonas communis* DSM 5604^T^	ASM436330v1	3.85	44.9	90.56	0.124	67.40	45.67
*Oceanospirullum linum* ATCC 11336^T^	MTSD02000001	3.78	49.13	90.21	0.153	69.20	52.34

*AF* alignment fraction, *ANI* average nucleic acid identity, *AAI* amino acid identity.

Examination of the component genes present in the ASxL5^T^ genome using the KEGG database^19^ revealed metabolic pathways typical of an aerobic *Gammaproteobacterium*. ASxL5^T^ contains a total of 75 genes assigned to bacterial motility proteins, including those involved in chemotaxis, flagella assembly and type IV pilus systems. Within the last category 9 out of 10 genes are responsible for twitching motility in a range of other organisms. The genome of ASxL5^T^ contained the complete ectoine biosynthesis pathway involved in the protective response to osmotic stress^20^, as might be expected for a halophilic organism. The genome also contains the complete pathways for many cofactors and vitamins including the riboflavin synthesis pathway. Hydrocarbon utilization pathways were incomplete although a gene for alkane 1-monooxygenase (alkB2) was present in ASxL5^T^. Homologues of genes identified as largely responsible for hydrocarbon degradation in *T. oleiverans* MIL-1^T^^21^ such as TOL_2658 (alkB) and TOL_2772 (alcohol dehydrogenase) were notably absent in the genome sequence of ASxL5^T^. A comparison of the distribution of genes in COG categories for ASxL5^T^ with *T. oleiverans* MIL-1^T^ is presented in Fig. 3B. Overall, the smaller genome of ASxL5^T^ contained proportionally less genes from each COG category compared to the larger related genomes. When the number of genes in each functional category are expressed as a percentage of the genome, differences were noted in the percentage of genes in the translation, ribosomal structure, and biogenesis categories together with the energy production and conversion functional categories, which comprise a greater percentage of the ASxL5^T^ genome than the same groups present  in the *T. oleiverans* MIL-1^T^ genome*.* In contrast, *T. oleivorans* MIL-1^T^ has a greater percentage of genes within the replication, recombination and repair, and transcription categories compared to the ASxL5^T^ genome. Interestingly the greatest difference in the contents of each of the functional categories for the two genomes was the number of unknown genes present in ASxL5^T^ (Fig. 3B). KEGG module enrichment analysis was carried out where each KEGG module represents a collection of manually defined functional units used for annotation and the biological interpretation of genome sequence data. A comparison of the distribution of genes in complete KOG module pathways for ASxL5^T^ with *T. oleiverans* MIL-1^T^ is presented in Fig. 3C. This analysis indicates that while ASxL5^T^ has complete pathways for sulfur and nitrogen metabolism but *T. oleiverans* MIL-1^T^ does not. In contrast *T. oleiverans* MIL-1^T^ has a complete pathway for cysteine and methionine metabolism that is incomplete in ASxL5^T^. Accordingly, ASxL5^T^ has a signature module (defined as a set of genes that can be used as a phenotypic marker such metabolic capacity or pathogenicity; https://www.genome.jp/kegg/module.html) for sulfate assimilation that is absent in *T. oleiverans* MIL-1^T^. Comparison of the gene content of ASxL5^T^ with a list of genes suggested to be indicative of a predatory lifestyle^3^ was inconclusive. Whilst the *waaL* gene which encodes a ligase associated with linking O-antigen polysaccharide to the core was present in the ASxL5^T^ genome (but is common in many Gram-negative bacteria), a tryptophan 2,3-dioxygenase (TDO) gene that may include a 60 amino acid region common in predatory bacteria^22^, was absent. Additional predatory signature genes included those that encode enzymes involved in the mevalonate pathway isoprenoid biosynthesis^3^ were also absent from the genome of ASxL5^T^. The transcriptional regulator gene *gnt*R was noted to be absent in the predator group examined^3^, but three *gnt*R-like genes could be identified in ASxL5^T^.

### Phenotypic comparison with related bacteria

The phenotypic characteristics of ASxL5^T^ are summarised in Table 3 and compared to the phenotypic characteristics reported in the literature for related genera^23–27^. Isolates from *T. marinus*, *T. olevorans, B. sanyensis* and *Oceanobacter kriegii* are motile, halotolerant, oxidase positive rods, but have few other phenotypic characteristics in common with ASxL5^T^. The average pH of the oceans is 8.1 (https://ocean.si.edu/ocean-life/invertebrates/ocean-acidification#section_77), a feature reflected in the basic pH survival range of *T. marinus*, *T. olevorans, B. sanyensis* and *O. kriegii*. ASxL5^T^ is adapted to a greater pH range (4–9) typical of non-marine species. The phenotypic characteristics of *Thalassolituus* sp. C2-1.are unknown. The growth temperature range of ASxL5^T^ was generally wider than the marine strains (4–42 °C), although some but not all isolates of *T. marinus* were thermotolerant. Further phenotypic characterisation was hampered by the inability to grow ASxL5^T^ in broth medium. Using API 20E tests with material scraped from BA plates, ONPG, arginine dihydrolase, lysine decarboxylase, ornithine decarboxylase, citrate utilisation, urease, tryptophan deaminase, gelatin hydrolase, tests were all negative, whilst indole, acetoin and H_2_S were not produced. Carbohydrates that were not fermented included: glucose, mannose, inositol, sorbitol, rhamnose, sucrose, melibiose, amygdalin and arabinose. The cellular fatty acid profiles of strain ASxL5^T^ compared to published related reference strains are shown in Table 4. The predominant cellular fatty acids were C16 : 1ω6c and/or C16 : 1ω7c, C16:0, and C18:1 ω9. Hydroxy fatty acids C12:0 3-OH and C10:0 3-OH were also present. The proportion of C16:0 was higher in ASxL5^T^ than the reported values for related genera. In contrast, there was a reduced proportion of C18 : 1ω7c and/or C18 : 1 ω6c in ASxL5^T^ compared to that reported for *T. marinus* IMCC1826^T^ *T. oleivorans* MIL-1^T^ and *O. kriegii* DSM 6294^T^ but not detected in *B. sanyensis* KCTC 32220^T^. A comparison to the fatty acid profiles of ASxL5^T^ with ASxLS revealed minor differences in quantities of individual fatty acids between the two strains, which is consistent with the genomic DNA sequences that they belong to the same species. Poly-3-hydroxybutyrate (PHB) granules were not detected using the Sudan Black test.

**Table 3:** Phenotypic characteristics of ASx5L^T^ and closest relatives.

Characteristic		ASxL5^T^	*T. marinus*	*T. oleivorans*	*B. sanyensis*	*O. kriegii*
Growth temperature (°C)	42	+	V	−	+	−
	37	+	V	−	+	NK
	25	+	+	+	+	+
	4	+	V	+	−	−
Catalase		+	+	V	+	+
Oxidase		+	+	+	+	+
PHB accumulation		−	−	−	+	+
pH range		4–9	6–9	7.5–9	6–10	5–9
Salt tolerance (%)		0.5–5	0.5–5	0.5–5	1–10	NK
Cell shape		CR	CR	CR	SR	SR
Mean cell dimensions (μm)	Length	1.6–2^a^	1.2–2.5	1.2–3.1	2.1–2.8	2.6–3.6
	Width	0.3–0.4	0.4–0.5	0.25–0.77	0.5–0.6	0.8–1.2

*V* varies according to strain, *NK* not known, *CR* curved rod, *SR* straight rod.

^a^Grown on BA agar without host. Data for reference strains were collated from a literature survey^23–27^.

**Table 4:** Fatty acid analysis of ASx5L^T^ and closest relatives.

Fatty acid	ASxL5^T^	*T. marinus* IMCC1826^T^	*T. oleivorans* MIL-1^T^	*B. sanyensis* KCTC 32220^T^	*O. kriegii* DSM 6294^T^
C10 : 0	2.3	Tr^b^	1.2	tr	tr
C12 : 0	2.3	1.5	1.4	8.6	21.2
C14 : 0	1.9	3.6	3.5	1	–
C16 : 0	25.3	14	13.3	13.2	13.4
C18 : 0	2.0	–	2.5	–	–
C17 : 1ω8c	1.3	1.2	–	3.8	–
C10 : 0 3-OH	2.3	3.7	3.6	4	11.2
C12 : 1 3-OH	4.7	6.3	6.5	2.9	6.7
**Summed features**^a^					
3	40.6	48	45.4	40.7	26.8
8	5.7	21.1	22.6	–	20.7

^a^Summed features represent groups of two fatty acids that could not be separated by GLC with the MIDI system. Summed feature 3 is C16 : 1ω6c and/or C16 : 1ω7c; summed feature 8 is C18 : 1ω7c and/or C18 : 1ω6c.

^b^tr, trace (< 1.0%). Data for reference strains were obtained from a literature survey^23–27^.

### ASxL5^T^ preys on ***Campylobacter*** species and other Gram-stain negative hosts

**Figure 4:**
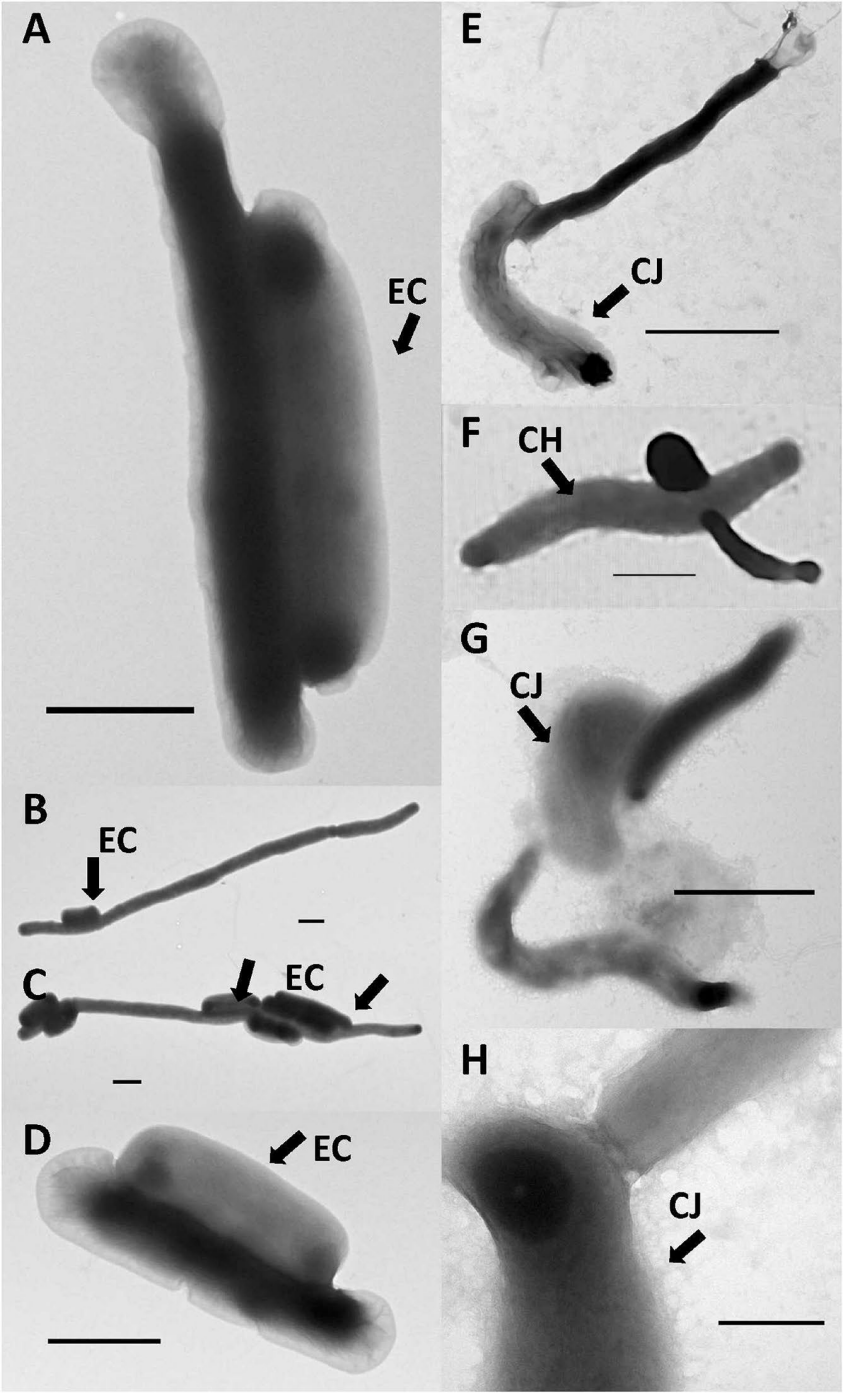
TEM of ASx5L^T^ interacting with prey showing: (**A**–**D**) with *E. coli* prey; (**E**–**H**) with *C. jejuni* prey. (**A**) typical cell ASx5L^T^ attached to a single *E. coli* (EC) cell; (**B**) filamentous ASx5L^T^ attached to single EC cell; (**C**) filamentous ASx5L^T^ cells attached to multiple EC cells; (**D**) smaller ASx5L^T^ cell attached to a single *E. coli* (EC) cell; (**E**) single ASx5L^T^ cell attached to a *C. jejuni* (CJ) cell; (**F**) ASx5L^T^ attacking a *C. hyointestinalis* (CH) cell; (**G**) two ASx5L^T^ cells attacking a CJ cell; (**H**) close view of attachment point of ASx5L^T^, close to apex of the CJ cell (bar 0.2 μm). Bar represents 1 μm in (**A**–**G**).

The predatory activity of the ASxL5^T^ bacterium was investigated to determine the prey range. The bacterium was able to form plaques on *Campylobacter* species including: *C. hyointesinalis* 11608^T^, *C. jejuni* PT14, *C. jejuni* 12662, *C. jejuni* NCTC 11168^T^; *C. coli* NCTC 12667; *C. helveticus* NCTC 12472; *C. lari* NCTC 11458 and *C. upsaliensis* NCTC 11541^T^. Testing of a wider selection of Gram-stain negative and Gram-stain positive bacteria, using cultures listed in the Host Range Determination section of Methods, revealed that ASxL5^T^ could also form plaques on *Escherichia coli* NCTC 86, *Citrobacter freundii* NCTC 9750^T^ and *Klebsiella oxytoca* 11466. TEMs of the interaction with *E. coli* NCTC 86 are shown in Fig. 4A–D whilst the interaction with *C. jejuni* PT14 and *C. hyointestinalis* S12 are shown in Fig. 4E–H. The attack mechanism appeared to differ between the prey types tested, with one or more *E. coli* cells becoming attached to each ASxL5^T^ cell, positioned laterally along the extended cell before adsorption. In contrast ASxL5^T^ appeared to attach to campylobacters via a single contact point, often with the predator cell apex, making contact near the *Campylobacter* cell apex (Fig. 4H).

## Discussion

Predatory bacteria have evolved to exploit abundant prey sources. It is becoming apparent that they are widespread in many different environments. The ASxL5^T^ bacteria was able to be isolated from slurry using phage isolation methods because of the narrow dimensions of members of the population. The genomic relatedness of ASxL5^T^ to members of the marine bacterial family *Oceanospirillaceae* was surprising, even though the organism was halotolerant being able to grow on 5% salt containing medium. Water quality analysis of the slurry revealed the sodium chloride level to be less than 0.1%. The slurry is therefore far from a marine environment—geographically and chemically. The presence of three related, but non-identical isolates from the same source, provided evidence that these predators were thriving in this non-marine environment. Moreover, microbiome analysis (data files available from https://www.ebi.ac.uk/ena/browser/view/PRJEB38990) revealed identical 16S rRNA gene sequences to be in the top 50 most abundant operational taxonomic units (OTUs) in the slurry over several sampling intervals. Several uncultured bacteria were identified in the Genbank database that had similar 16S rRNA gene sequences to the ASxL5^T^ bacterium. These sequences together with those of ASxL5^T^, ASxS5 and ASxO5 appeared to represent a distinct clade separated from *Thalassolituus* and *Oceanobacter* (Fig. 2). Three of the uncultured bacteria (GQ921362, GQ921357 and GQ921396) were all isolated from fracture water, from a depth of 1.3 km in a South African gold mine in 2009, while a further two (DQ256320 and DQ337006) were obtained from subsurface water (also in South Africa) in 2005. The most closely related 16S rRNA gene sequence relative to ASxL5^T^ is a partial 16S rRNA gene sequence that was obtained from enrichment culture of sandy sediment, obtained from a beach in Northern France in 2006 (accession number AM292408^28^). A further closely related 16S rRNA gene sequence from an uncultured bacterium, HQ183822.1, was obtained from a collection pool leached from a municipal landfill site in China^29^. Clearly the ASxL5^T^ bacteria is not highly represented in taxonomic databases but it is likely that these sequences from uncultured bacteria represent similar organisms to ASxL5^T^, which are distributed worldwide, often in challenging environments. The closest relatives to ASxL5^T^ from whole genome phylogenetic analysis were: *Thalassolituus* sp. C2-1, *T. marinus*, *T. oleivorans*. and *O. kriegii*^23–27^. *Thalassolituus* are members of the marine obligate hydrocarbonoclastic bacteria (OHCB) and are prevalent in marine and terrestrial environments often becoming dominant following incidents of hydrocarbon pollution^30,31^. *Oceanobacter* are not members of the OHCB group but are isolated from marine environments^32^.

The phenotypic data indicate that ASxL5^T^ is a novel species and a member of a previously unrecognised genus within the family *Oceanospirillaceae*. There are at present no unambiguous criteria for assignment of a newly isolated strain into a new genus. Attempts have been made to identify a universal genus boundary, for example that based on the genomic percentage of conserved proteins (POCP), with a suggested cut off value of 50% identity to reference strains^33^. Others have suggested using AAI values, which have an advantage over POCP in that they can be obtained from incomplete genomes^34^. The authors suggested that a strain is a representative of a different genus if the AAI value is less than 74% when compared with the type strain of the type species. The type genus in the family *Oceanospirillaceae,* is *Oceanospirillum* and the type strain is *O. linum* ATCC 11336^T^. The AAI value between ASxL5^T^ and *O. linum* ATCC 11336^T^ is 54.34% and between ASxL5^T^ and *T. oleivorans* MIL-1^T^ (genus type strain) is 67.61% indicating that ASxL5^T^ represents a novel genus distinct from *Thalassolituus*. Using the 16S rRNA gene sequences as the taxonomic criteria with a suggested genus delimitation boundary of 94.5%^35^ would potentially place ASxL5^T^ within the genus *Thalassolituus* exhibiting a 16S rRNA sequence identity of 95.03% with *T. oleivorans* MIL-1^T^ and 96.17% to *T. marinus* IMCC1826^T^. However, it would also be placed in the *Bacterioplanes* genus with a 16S rRNA gene identity with *B. sanyensis* NV9 of 94.64% illustrating that the use of a single gene such as the16S rRNA gene, can lead to arbitrary taxonomic assignments. Another suggested method uses both ANI and genome alignment fraction (AF) to examine the clustering of data points from all the type and non-type strains of existing genera^36^. The authors suggest the use of a genus demarcation boundary in conjunction with the estimated genus inflection point that is specific to the taxon that is being analyzed. However, without sufficient complete genome sequences from *Thalassolituus* isolates it is not possible to determine whether ASxL5^T^ belongs to the *Thalassolituus* genus by this method. The whole genomic phylogenic tree should be interpreted with discretion due to the limited availability of complete genome sequences to carry out the analysis and secondly methods for whole genome comparison do not account for the substantial difference in the sizes of the genomes being compared. They measure the similarity of core single-copy genes that are conserved between related genera but do not take into account the very large number of genes which are not present in the much smaller genome of ASxL5^T^. Clearly ASxL5^T^ and the group including *Thalassolituus*, *Oceanobacter* and *Bacterioplanes* have a common ancestor, but evolution has taken a different path resulting in genome reduction, possibly as an adaption to a predatory lifestyle. This contrasts with *T. oleivorans* MIL-1^T^ that is 28% larger, and which has evolved under different environmental pressures to utilise hydrocarbons^23,30^. An interesting comparison can be made to obligate intracellular parasites and symbionts such as *Rickettsia, Chlamydia* and *Buchnera* which have genome sizes of approximately 1 Mb, having undergone significant evolutionary genome degradation as the ability to exploit host-cell metabolites leads to gene loss^37^. An evolutionary change from a marine chemolithotroph to predatory lifestyle could result in a similar genome size reduction. COG and KEGG analysis highlighted global differences in the numbers of genes devoted to specific functions and in the genomic pathways between ASxL5^T^ and *T. oleivorans* MIL-1^T^ and are not due to the extensive acquisition of mobile genetic elements. The difference in the G + C ratios for the whole genomes of ASxL5^T^ of 56.1% and *T. oleivorans* MIL-1^T^ of 46.6% is also indictive of genus separation.

Examination of the coding content of the ASxL5^T^ genome provided functional insights into the phenotypic characteristics. The presence of genes that encode type IV pili (Tfp) are of particular interest as these facilitate cell movement referred to as social gliding or twitching without flagella over surfaces. Tfp are reported to have other functions including predation, pathogenesis, biofilm formation, natural DNA uptake, auto‐aggregation of cells and development^38^. That the ASxL5^T^ genome contains 18 genes encoding diguanylate cyclase (enzyme that catalyses the conversion of 2 guanosine triphosphate to 2 diphosphate and cyclic di-GMP) and 6 genes encoding the corresponding diguanylate cyclase phosphodiesterase (catalyses the degradation of cyclic di-GMP to guanosine monophosphate) is of interest because cyclic-di-GMP is an important second messenger involved in processes that include biofilm development and detachment, motility, cellular attachment and virulence^39,40^. It should also be noted that in *Bdellovibrio bacteriovorus* cyclic-di-GMP has been shown to control the switch between free-living and predatory lifestyles^41^.

Most research into predatory bacteria has centred on *Bdellovibrio*, *Bdellovibrio*-like organisms and *Myxocococcus* species. These and other known examples of predatory bacteria form a taxonomically diverse group. Despite this diversity, a group of signature protein families that reflect the phenotypes of 11 known predatory bacteria have been identified^3,22^. However, only the gene encoding O-antigen ligase (waaL) was identified, which is notably prevalent in Gram-negative bacteria. This form of analysis was not helpful for the assignment of ASxL5^T^ as a predator, likely because of the novel attack strategies it employs. The availability of more diverse predatory bacterial genomes will enable the development of finer resolution analyses that take into account evidence of functional and environmental differences between group members. Examples of predatory bacteria not included in this analysis include *Cupriavidus necator*^42^ and members of the Bradymonabacteria^43^ as more predatory taxa are established as researchers investigate different microbial communities.

**Figure 5:**
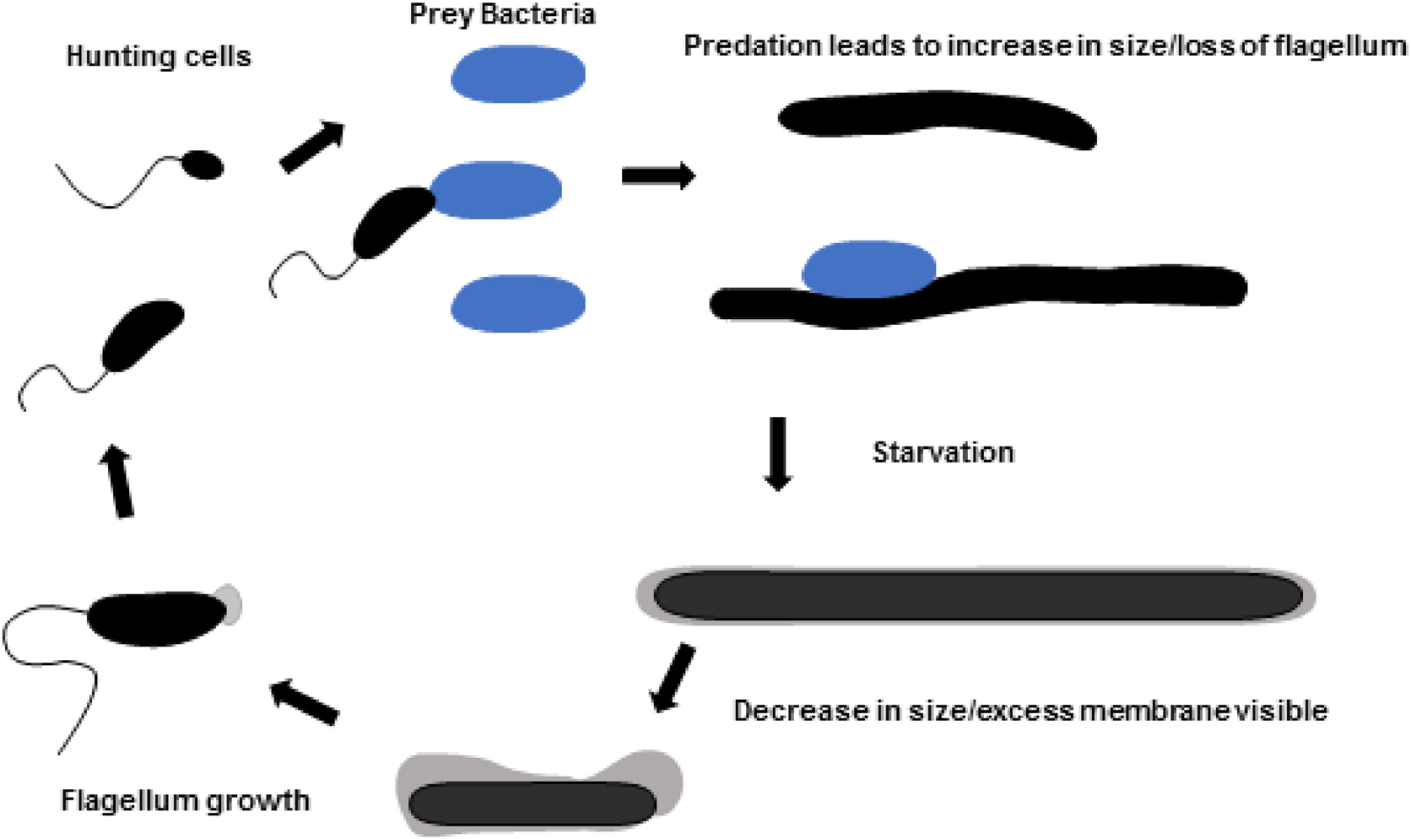
Proposed life cycle of the ASxL5^T^ bacterium.

The most remarkable features of the ASxL5^T^ bacterium as captured by TEM images, are its unique flexible morphologies that facilitate interactions with prey bacteria. The type of interaction observed is different from other predatory bacteria and has not been identified or reported previously. A proposed predatory life cycle of ASxL5^T^ is shown in Fig. 5. There are few examples in the literature of similar apical structures to those we report here, but these include those of *Terasakiispira papahanaumokuakeensis* an *Oceanospirillaceae* bacterium, that shows occasional apical enlargement^44^, and the Alphaproteobacteria, *Terasakiella pusilla* previously in the genus *Oceanospirillum*, that exhibits what are described as “polar membranes”^45^. The presence of coccal forms in older cultures is a frequent observation particularly for bacteria with curved morphology, such as *Vibrio*, *Campylobacter* and *Helicobacter*^46–48^, which probably represents a degenerative state. Further work is required to elucidate the precise life cycle of the ASxL5^T^ bacterium. to determine how it traps and feeds on its prey, and whether its genome encodes bioactive compounds that can be exploited for medicinal or biotechnological purposes.

**Description of *Venatorbacter* gen. nov.** *Venatorbacter* (Ven.a.tor, ba’c.ter, L. composed of venator from L. n. *venator*, ‘hunter’ and Gr. n. bacter, ‘a rod’. Venatorbacter, ‘a hunting rod’. Cells are aerobic, halotolerant, curved Gram-stain negative, motile rods. Catalase and oxidase activities are positive. Does not accumulate PHB. Growth is obtained at a range of temperatures from 4 to 42 °C. The pH range of 4–9 is unusual in the *Oceanospirillaceae* as most are intolerant of acid pH. The major fatty acids are C16 : 1ω6c and/or C16 : 1ω7c, C16:0, and C18:1 ω9; C12:0 3-OH and C10:0 3-OH are found as hydroxy fatty acids. Growth does not occur in broth medium. The DNA G + C content is 56.1 mol%. Members of this genus exhibit predatory behaviour with *Campylobacter* species and members of the *Enterobacteriaceae*. The phylogenetic position of the genus is in the family.

*Oceanospirillaceae* in the class *Gammaproteobacteria*. The type species is *Venatorbacter cucullus.*

**Description of *Venatorbacter cucullus* sp. nov.** *Venatorbacter cucullus* (cu'cull.us.; L. n. *cucullus* meaning cowl).

In addition, the description features of the genus, cells are of 1.63 µm in length by 0.37 µm wide when grown on BA or BHI. Colonies on BHI agar are small reaching 2 mm in diameter after 72 h. They are beige, translucent, circular, convex and shiny. Members of the species can use *E. coli*, *Klebsiella* spp. *Campylobacter* spp. and several other Gram-stain negative bacteria as prey.

The type strain ASxL5^T^ was isolated in Nottinghamshire UK from bovine slurry and is deposited at National Collection of Type Cultures (UK): accession number NCTC 14397 and the Netherlands Culture Collection of Bacteria (NCCB) accession number NCCB 100775. The complete genome sequence of ASxL5^T^ has been deposited at Genbank under accession CP046056.

## Methods

### Isolation and phenotypic characterisation of ASx5L^T^

The ASxL5^T^ bacterium was isolated from cattle slurry using a technique for phage isolation^9,49^. The slurry was diluted in 1:9 (w/v) in SM buffer (50 mM Tris–HCl [pH 7.5], 0.1 M NaCl, 8 mM MgSO4·7H2O, and 0.01% gelatine; Sigma Aldrich, Gillingham,UK) then incubated at 4 °C for 24 h with slow rotation to elute the predator into the buffer. The suspension was centrifuged at 3000*g* for 3 min. The supernatant was collected and subjected to a second centrifugation step for 5 min at 13,000*g*. The supernatant was then passed through a 0.45 µm membrane filter (Minisart; Sartorius, Gottingen, Germany) and a 0.2 µm membrane filter (Minisart) to remove any remaining bacterial cells. ASxL5^T^ was able to pass through these filters. Soft agar lawns of an isolate of *C. hyointestinalis* S12, (NCBI accession number CP040464) from the same slurry, were prepared using standard techniques^49^. The filtered slurry was dispensed as 10 µl droplets in triplicate, on each of these host cell plates and allowed to dry. The plates were incubated at 37 °C under microaerobic conditions (5% O_2_, 5% H_2_, 10% CO_2_, and 80% N_2_) in microaerobic jars for 48 h. Visible plaques obtained were extracted into SM buffer and transferred to fresh lawns of *C. hyointestinalis* S12 to propagate the lytic organism further. Once it was established that a bacterium was responsible for the lytic plaques rather than a bacteriophage, attempts were made to cultivate the organism independently from the host and characterize it further. Weak growth that improved on subculture was obtained on Brain Heart Infusion Agar (BHI; CM1136, Oxoid, Basingstoke, UK) and Horse Blood Agar No 2 (BA; CM0271 Oxoid) with 5% v/v defibrinated horse blood (TCS Biosciences Lt, Buckingham, UK, added) by aerobic incubation at 37 °C. Antimicrobial sensitivity testing was performed using the disk diffusion methods in accordance with the National Committee for Clinical Standards guidelines^50^. BHI agar incubated aerobically at 37 °C using discs with the following antibiotics (Oxoid): amoxycillin and clavulanic acid 30 µg; cefotaxime 30 µg; streptomycin 10 µg; ciprofloxacin 5 µg; ceftazidime 30 µg nalidixic acid 30 µg; imipenem 10 µg; azithromycin 15 µg; chloramphenicol 30 µg; cefoxitin 30 µg; tetracycline 30 µg; nitrofurantoin 300 µg; aztreonam 30 µg; ampicillin 10 µg; cefpodoxime 10 µg; trimethoprim-sulfamethoxazole 25 µg. Salt tolerance was established by cultivation aerobically at 37 °C on BHI agar plates to which additional NaCl was added to give a range of concentrations up to 10% w/v. The pH range was determined by cultivation aerobically at 37 °C on BHI agar plates where the pH range had been adjusted to be between 4 and 9 with either sterile HCl or sterile NaOH, and the target pH verified before pouring the plates. For cellular fatty acid analysis, ASxL5^T^ was cultured on BHI agar for 3 days, aerobically at 37 °C. The cellular fatty acids were extracted, prepared, and analysed according to the standard protocol of MIDI (Sherlock Microbial Identification System, version 6.10) by FERA Science Ltd, (York, UK).

### Microscopy

For TEM, ASxL5^T^ was cultured aerobically by spreading uniformly on BA for 24 h at 37 °C and harvested into 1 ml of 3% (v/v) glutaraldehyde in 0.1 M cacodylate buffer, fixed for 1 h at room temperature then centrifuged at 10,000*g* for 3 min. The pellet was then re-suspended gently into 600 μl of 0.1 M cacodylate buffer. The fixed ASxL5^T^ suspension was transferred onto Formvar/carbon film on copper 200 mesh grids. The bacteria stained with 0.5% (w/v) uranyl acetate for 1 min and examined by TEM using a TEI Tecnai G2 12 Biotwin microscope. The predator prey interaction was also examined by TEM as described above combining equal numbers of prey and predator in NZCYM broth (BD Difco™, Fisher Scientific UK Ltd, Loughborough) and incubating for 48 h at 37 °C, under microaerobic conditions for *Campylobacter* or aerobic conditions for *E. coli.* Prey and predatory bacteria were examined independently to establish any changes in cell morphology arising as a consequence of predation. Light microscopy for PHB accumulation was carried out using the Sudan Black method^51^.

### Host range determination

Overnight cultures of ASxL5^T^ were grown by spreading growth on BHI or BA plates using a sterile swab. The ASxL5^T^ cells were collected and suspended in MRD (CM0733, Oxoid) and then placed at 4 °C for 7 days, to starve the cells. NCTC reference or laboratory stock bacteria cultures were inoculated into BHI broth or Nutrient Broth No 2 (CM007, Oxoid), incubated overnight, centrifuged at 13,000*g* and re-suspended in MRD to an OD_600_ of 0.4. The cultures were: *Bacillus subtilis* NCTC 3610^T^, *Citrobacter freundii* NCTC 9750^T^, *Enterobacter aerogenes* NCTC 10006^T^*, Enterococcus faecalis* NCTC 775^T^, *Escherichia coli* NCTC 86, *Klebsiella oxytoca* 11466, *Leuconostoc mesenteroides* NCTC 10817, *Listeria monocytogenes* NCTC 4885, *Paenibacillus macerans* NCTC 6355^T^, *Providencia stuartsii* NCTC 10318, *Pseudomonas fluorescens* SMDL, *Rhodococcus hoagie* NCTC 1621^T^, *Salmonella enterica* Montevideo NCTC 5747, *Serratia liquefaciens* NCTC 10861, *Staphylococcus aureus* NCTC 8532^T^, *Streptococcus pneumoniae* NCTC 7465^T^, *Yersinia enterocolitica* NCTC 10460. *Campylobacter* hosts were incubated microaerobically at 37 °C on BA plates and then suspended in NZCYM broth. *Campylobacter* hosts tested were: *C. coli* 12667 NCTC, *C. jejuni* 12662, *C. jejuni* PT14, *C. jejuni* NCTC 11168^T^, *C. helveticus* NCTC 12472, *C. lari* NCTC 11458, *C. upsaliensis* NCTC 11541^T^, *C. hyointestinalis* NCTC 11608^T^. Cells were collected in MRD, centrifuged at 13,000*g* and re-suspended in MRD to an OD_600_ of 0.4. An aliquot of 0.5 ml of the suspensions was added to 5 ml aliquots of molten NZCYM top agar (0.6% agar) and poured on to 1.2% NZCYM baseplates. Once set and dried, serial dilutions of ASxL5^T^ were dispensed as 20 µl droplets in triplicate onto each lawn plate. The incubation temperature and atmosphere were dependent on the test bacteria’s requirements.

### 16S rRNA gene and whole genome sequence determination

DNA was prepared from bacterial isolates using GenElute™ Bacterial Genomic DNA Kit (Sigma Aldridge). PCR amplification of 16S rRNA gene and sequence determination of the product using dye-terminator chemistry (Eurofins Value Read Service, Germany) was carried out using standard methods. The sequences were compared with other 16S rRNA gene sequences using the BLAST-N program to identify and collect closely related species. These were aligned using ClustalW within the MEGA X program^52^. Phylogenetic trees were reconstructed using MEGA X using the Maximum Likelihood method based on the Tamura-Nei model^53^ with 1000 bootstrap replications^54^. DNA for whole genome sequencing was extracted using the PureLink™ Genomic DNA Kit (Fisher Scientific, Loughborough, UK). The genome sequence of ASxL5^T^ was determined using a combination of Illumina MiSeq consisting of 250 bp paired-end reads using libraries prepared from the Nextera tagmentation kit, and long reads of 2 to 20 kb from the PacBio (Pacific Biosciences) platform performed at the Nu-Omics DNA Sequencing Research Facility, Northumbria University. The genome was assembled using CLC Genomics Workbench 12.0.3 (Qiagen, Aarhus, Denmark). ASxL5^T^ cultures were deposited at National Collection of Type cultures (UK) and the Netherlands Culture Collection of Bacteria (NCCB). Genomes of related organisms used for comparisons were: *Thalassolituus oleivorans* MIL-1^T^ (accession HF680312, complete); *Bacterioplanes sanyensis* KCTC 32220^T^ (accession BMYY01000001, incomplete); *Oceanobacter kriegii* DSM 6294^T^ (accession NZ_AUGV00000000, incomplete); *Marinomonas communis* DSM 5604^T^ (accession ASM436330v1, incomplete), *Oceanospirullum linum* ATCC 11336^T^ (accession MTSD02000001, incomplete) and *Thalassolituus* sp. C2-1 (accession NZ_VNIL01000001, incomplete). The alignment fraction (AF) and average nucleic acid identity (ANI) were determined using the JGI Genome Portal^36^ hosted at https://img.jgi.doe.gov//cgi-bin/mer/main.cgi?section=ANI&page=pairwise. Amino acid identities (AAI) were determined using the method of Rodriguez-R & Konstantinidis^55^. An estimated maximum-likelihood phylogenetic tree was generated with GToTree 1.5.54^11–18^. Input genomes representing available reference genomes were selected from reference genera identified as related to ASxL5^T^ from the 16S rRNA phylogeny. The tree was annotated using the Interactive Tree Of Life online tool, (https://itol.embl.de/). Functional annotation and analysis of the ASxL5^T^ genome was carried out using KEGG (Kyoto encyclopedia of genes and genomes) module enrichment assignment using the BlastKOALA KEGG online tool^19^. The distribution of COG categories (clusters of orthologous groups) was determined using the eggNOG-mapper online tool^56^.

## Data Availability

Accession code: Genome sequence of ASxL5^T^ CP046056.
